# MicroRNA528 and Its Regulatory Roles in Monocotyledonous Plants

**DOI:** 10.3390/ijms26157334

**Published:** 2025-07-29

**Authors:** Hailin Fu, Liwei Zhang, Yulin Hu, Ziyi Liu, Zhenyu Wang, Fafu Shen, Wei Wang

**Affiliations:** College of Agronomy, Shandong Agricultural University, No. 61 Daizong Street, Tai’an 271018, China

**Keywords:** MicroRNAs, miR528, post-transcriptional gene regulation, plant development, stress

## Abstract

MicroRNA528 (miR528) is a microRNA found only in monocotyledonous (monocot) plants. It has been widely reported that miR528 is involved in the regulation of plant growth and development, such as flowering, architecture, and seed and embryogenic development, in addition to playing a crucial role in response to various biotic and abiotic stresses, such as plant pathogens, salt stress, heat/cold stress, water stress, arsenic stress, oxidative stress, heavy-metal stress, and nutrient stress. Given that it is specific to monocot plants, to which the major staple food crops such as rice and wheat belong, a review of studies investigating its diverse functional roles and underlying mechanisms is presented. This review focuses on the processes in which miR528 and its targets are involved and examines their regulatory relationships with significant participation in plant development and stress responses. It is anticipated that more biological functions and evolutionary effects of miRNA targets will be elucidated with the increase in knowledge of miRNA evolution and examination of target mRNAs.

## 1. Overview of Role of miRNAs in Plant Development and Stress Responses

MicroRNAs (miRNAs) are a class of single-stranded non-coding small RNAs (sRNAs) with a mature sequence length ranging from 20 to 24 nucleotides [1]. In plants, miRNAs are processed from larger precursor molecules that are transcribed from miRNA genes (*MIRs*) by RNA polymerase II (Pol II). The precursor molecules form self-complementary stem loop structures and are processed into miRNA:miRNA-star duplexes by a dicing complex, including DICER-LIKE 1 (*DCL1*), HYPONASTIC LEAVES 1 (*HYL1*), and SERRATE (*SE*). The miRNA:miRNA-star duplexes are 2′-O-methylated at the 3′ ends by the methyltransferase HUA ENHANCER 1 (*HEN1*) within the nucleus and are exported into the cytoplasm, where they are incorporated into RNA-induced silencing complex (RISC) by HASTY (*HST*), which is a member of the importin β family of nucleocytoplasmic transporters. Upon RISC loading, the mature, methylated miRNA is complexed with Argonaute 1 (*AGO1*), while the miRNA-star strand is removed and degraded [2]. The mature miRNA functions to convey sequence-specific negative regulation of endogenous or exogenous target mRNAs by degradation or translation repression through sequence complementarity at the post-transcriptional level [3,4].

In the past two decades, miRNAs have been increasingly recognized for their involvement and the pivotal roles they play in regulating plant growth, development, and responsiveness to various environmental stress conditions [5,6,7]. For example, wheat (*Triticum aestivum* L.) miRNA *tae-miR408* was found to determine the heading time by mediating the expression of TIMING OF CAB EXPRESSION-A1/B1/C1 (*TaTOC1s*), which is known as a key component of the plant circadian clock [8]. It was also demonstrated that *tae-miR408* can mediate plant responses to phosphate (Pi) starvation and salt stress by regulating the expression of a suite of six genes that are associated with Pi acquisition and ABA signaling transduction [9]. On the other hand, transgenic expression of short tandem target mimic (STTM), which targets genetic downregulation of 35 miRNA families in rice (*Oryza sativa* L.), resulted in hereditable alterations in a number of agronomic traits, including plant height, tiller number, and grain number [10]. Among these targeted miRNAs, *osa-miR398* plays a crucial role in determining panicle length, grain number, and grain size in rice [10]. In addition, it positively regulates rice basal defense against the blast fungus *Magnaporthe oryzae* by boosting hydrogen peroxide (H_2_O_2_) production through the regulation of *CSD1* and *CSD2*, which encode Cu/Zn superoxide dismutases (SODs), and *CCSD*, a copper chaperone for SOD [11]. However, *osa-miR398* exhibits a negative regulatory role in plant defense in other species, such as *Arabidopsis thaliana* [12] and barley [13].

A striking feature of *miR528* in monocotyledonous plants is the remarkable diversity of its expression patterns and biological functions across different species. Numerous studies have demonstrated that *miR528* exhibits species-specific regulatory roles, with some even reporting contrasting expression levels and functional outcomes among different monocots. Despite such variations, *miR528*, as a monocotyledon-specific microRNA (miRNA), plays a significant regulatory role in plant growth, development, and responses to environmental stresses. For example, *miR528* is reportedly involved in plant development processes, such as flowering [14,15], plant architecture [16], and seed and embryogenic development [17], in addition to mediating plant responses to various biotic and abiotic stresses, such as plant pathogens [18], salt stress [16], heat/cold stress [19], water stress [20], arsenic (As) stress [21], oxidative stress [22], heavy-metal stress [23], and nutrient stress [24]. Understanding the *miR528*-mediated regulatory network would undoubtedly highlight miRNA studies as a fruitful avenue for further investigations that may have far-reaching implications in studying molecular regulation of important agronomic traits and plant defense in monocot crops and provide a set of new gene tools for crop improvements by marker-assisted breeding and biotechnology. Here we attempt to provide a synopsis of the growing recognition that *miR528* is a significant player in modulating plant growth, development, and stress responses, imparting a solid knowledge base for further investigations on the more defined biological functions and evolutionary aspects of *miR528* and its molecular targets.

## 2. *miR528* and Its Targets

### 2.1. MIR528 Genes in Monocots

**Figure 1:**
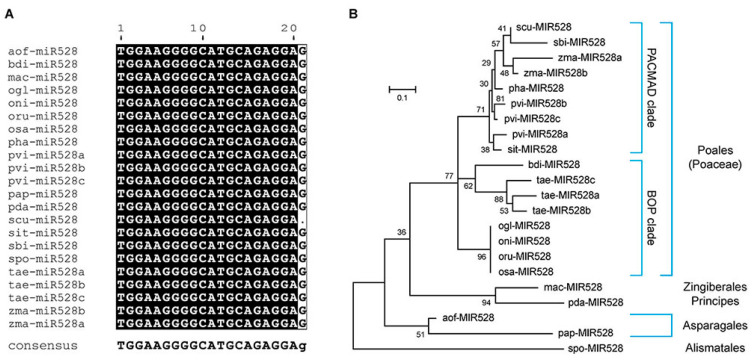
The sequence alignment and phylogenetic analysis of *miR528*. (**A**) Alignment of mature *miR528* sequences. *miR528* derived from 17 monocot plant species, including *Asparagus officinalis* L. (aof), *Brachypodium distachyon* (L.) *P*.Beauv. (bdi), *Musa acuminata* Colla (mac), *Oryza glaberrima* Steud. (ogl), *Oryza nivara* S.D.Sharma and Shastry (oni), *Oryza rufipogon* Griff. (Oru), *Oryza sativa* L. (osa), *Panicum hallii* Vasey (pha), *Panicum virgatum* L. (pvi), *Phalaenopsis aphrodite* Rchb.f. (pap), *Phoenix dactylifera* L. (pda), *Saccharum* hybrid cultivar (scu), *Setaria italica* (L.) *P.* Beauvois (sit), *Sorghum bicolor* (L.) Moench (sbi), *Spirodela polyrhiza* (L.) Schleid. (spo), *Triticum aestivum* L. (tae), and *Zea mays* L. (zma). The black-shaded blocks indicate highly conserved residues. (**B**) The phylogenetic tree reconstructed with precursors of *miR528*. We aligned the *miR528* precursor sequences of all the 17 monocot plant species using Molecular Evolutionary Genetics Analysis (MEGA) version 6.06 software [31] with MUltiple Sequence Comparison by Log-Expectation (MUSCLE) and reconstructed the phylogenetic tree using the maximum likelihood (ML) method using the general time reversible (GTR) substitution model with the default set of gamma distribution among-site rate variation. To compare the similarities and differences of the ML phylogenetic tree, we also reconstructed the phylogenetic tree with the maximum parsimony (MP) method using MEGA (Supplementary Figure S1). The phylogenetic tree of the 17 monocot plants used in the study was gathered from Monocots Plant Annotated Genomes Database (PLAZA) 4.5 (https://bioinformatics.psb.ugent.be/plaza/, accessed on 6 December 2024) [32]. The 17 monocot plants belong to five orders of the Liliopsida. The five orders are as follows: Alismatales, Asparagales, Principes, Zingiberales, and Poales. Poaceae is a large and nearly ubiquitous family of monocotyledonous flowering plants known as grasses, containing two sister lineages (or clades): the Panicoideae, Arundinoideae, Chloridoideae, Micrairoideae, Aristidoideae, and Danthonioideae (PACMAD) clade and the Bambusoideae, Oryzoideae, and Pooideae (BOP) clade. The name of the PACMAD clade comes from the first initials of the six included subfamilies, i.e., Panicoideae, Arundinoideae, Chloridoideae, Micrairoideae, Aristidoideae, and Danthonioideae [33]. The BOP clade contains three subfamilies from whose initials its name derives: bamboos (Bambusoideae); Oryzoideae, including rice; and Pooideae, including important cereal crops such as wheat [34].

Since its first discovery in rice [25], *miR528* has been identified in many other monocot plants, such as wheat [26], maize [27], sorghum [28], and sugar cane [29]. To facilitate the investigations of characteristic features of *miR528* loci (*MIR528*), we extracted the *miR528* information from 17 monocot plant species in the Plant miRNA ENcyclopedia (PmiREN; version 2.0) database (http://www.pmiren.com/, accessed on 20 January 2025) [30]. As shown in Table 1, apart from maize (*Zea mays* L.), which has two members, and switchgrass (*Panicum virgatum* L.) and wheat (*Triticum aestivum* L.), which have three members, *miR528* is encoded by a single gene. The precursors of *miR528* are endowed with a well-developed stem-loop structure (Supplementary Figure S1), and their mature sequences are identical, except for *scu-miR528* derived from the *Saccharum* hybrid cultivar, which differs from the others by a single nucleotide at the 3′ end (Figure 1A).

To explore the evolutionary history of *miR528* in monocots, we reconstructed a phylogenetic tree of the *miR528* precursors. The presence of *MIR528* can be traced back as early as in an Alismatales duckweed species (*Spirodela polyrhiza* L. Schleid) but not in any eudicots, gymnosperms, or basal angiosperms (Figure 1B and Figure S1). It is plausible that *miR528* emerged after the split of monocots from their common ancestor with eudicots, and the phylogenetic relationships of *miR528* homologs in each subgroup were consistent with plant speciation and evolution (Figure 1B and Supplementary Figure S1). It has recently been revealed that MIR528 genes were found in a syntenic block of asparagus (*Asparagus officinalis* L.), pineapple (*Ananas comosus* L.), and rice, but such a syntenic relationship was lost in banana (*Musa acuminata* Colla) [19]. In general, our analysis of the updated PmiREN database confirms that *miR528* is a miRNA restricted to monocots.

### 2.2. Targets of miR528

Many studies indicate that the target genes of *miR528* may have undergone high levels of divergence [16,18,35]. To further reveal the target genes of *miR528*, we performed genome-wide search and prediction of miR528 targets in the five grain or forage crop plants with economic significance, including *O. sativa*, *Z. mays*, *T. aestivum*, *Setaria italica*, and *P. virgatum* using the plant small RNA target analysis server (psRNATarget) web server [36], in combination with relevant public degradome sequencing data that were gathered from the PmiREN database. As a result, 19, 23, 13, 14, and 22 putative target genes of *miR528* were predicted in *O. sativa*, *Z. mays*, *T. aestivum*, *Setaria italica*, and *P. virgatum*, respectively (Supplementary Table S2). The putative target genes of *miR528* showed both structural conservation and specificity among the five examined plant species.

**Figure 2:**
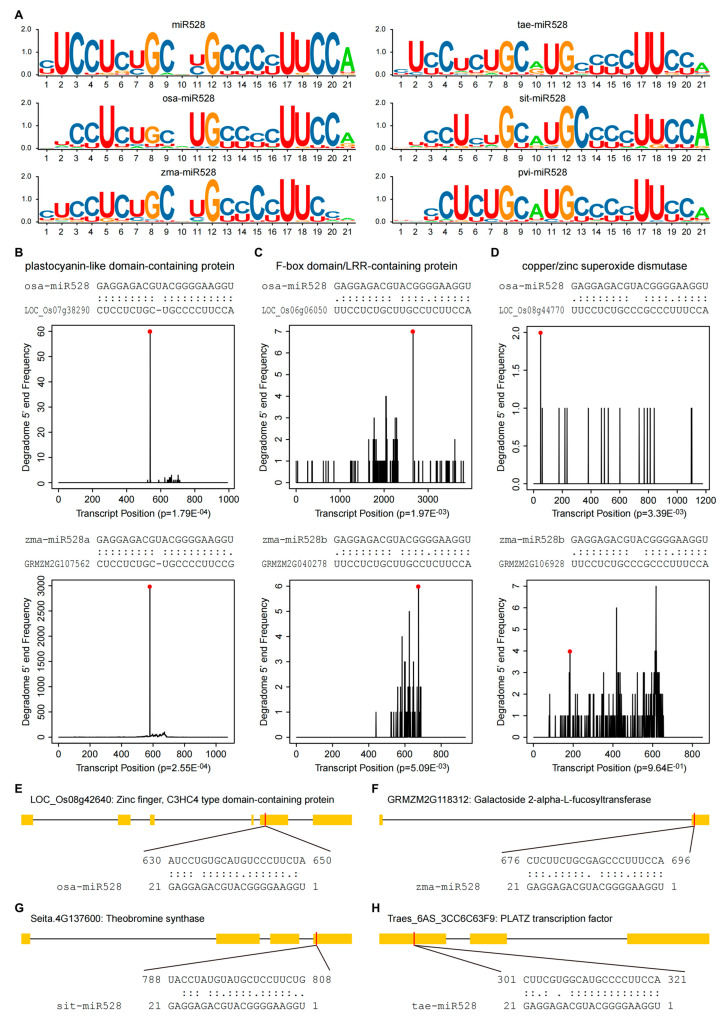
The target genes of *miR528*. (**A**) The sequence logos of *miR528* target sites. Toolkit for Biologists integrating various biological data handling tools (TBtools) was used to draw the sequence logos (https://github.com/CJ-Chen/TBtools/, accessed on 25 December 2024) [38]. The sequence logos of *miR528* target sites are conserved in rice, corn, wheat, millet, and switchgrass. These conserved target genes encode three types proteins, including plastocyanin-like domain-containing proteins, F-box domain- and (or) leucine-rich repeat (LRR)-containing proteins, and the copper/zinc superoxide dismutase. *osa-miR528*, *zma-miR528*, *tae-miR528*, *sit-miR528*, and *pvi-miR528* represent the sequence logos of all the predicted *miR528* target sites in rice, corn, wheat, millet, and switchgrass, respectively. (**B**–**D**) The conserved target genes of *miR528*. The target genes of *miR528* were predicted by searching the transcripts for complementary sequences using the psRNATarget server with a more stringent cut-off threshold: maximum expectation (**E**) = 3.0 (https://www.zhaolab.org/psRNATarget/, accessed on 26 December 2024) [36]. Degradome sequencing data were downloaded from the PmiREN database. The upper parts of B-D present the alignments of *miR528* with its target sequences, and the lower parts are the frequency of the 5′ end of the degradome tags within the full-length target transcripts. The solid lines indicate matched RNA base pairs. One dot shows G-U mismatch, and two dots represent other types of mismatch. (**B**) LOC_Os07g38290 and GRMZM2G107562, encoding a plastocyanin-like domain-containing protein, are the targets of *miR528* in rice and maize, respectively; (**C**) LOC_Os06g06050 and GRMZM2G040278, encoding F-box domain- and (or) LRR-containing proteins, are the targets of *miR528* in rice and maize, respectively; (**D**) LOC_Os08g44770 and GRMZM2G106928, encoding a copper/zinc superoxide dismutase, are the targets of *miR528* in rice and maize, respectively. (**E**–**H**) Examples of non-conserved target genes of *miR528* predicted in different monocot species, including genes involved in flowering regulation, glycosylation, alkaloid biosynthesis, and transcriptional regulation. The black line represents intron, the rectangle filled yellow represents exon, and miRNA complementary sites (red) are shown. The RNA sequences of each complementary site from 5′ to 3′ and the *miR528* sequence from 3′ to 5′ are shown in the expanded regions.

The conserved genes that are targeted by *miR528* encode three types of proteins, including plastocyanin-like domain-containing proteins (PLC), proteins containing the F-box domain and (or) leucine-rich repeat (LRR), and enzymes related to reactive oxygen species (ROS) homeostasis (Supplementary Table S2). The predicted target genes of *miR528* in both rice and maize include all the three types of proteins mentioned above (Figure 2B–D), indicating that *miR528* has conserved targets across different monocotyledonous species. The ROS homeostasis-related proteins include polyphenol oxidase (PPO), ascorbate oxidase (AAO), amine oxidase (AO), SOD, and peroxidase (POD), which are involved in both ROS generation and scavenging. As both the plastocyanin-like domain-containing proteins and the ROS homeostasis-related proteins are copper-containing proteins, *miR528* is also regarded as a copper miRNA (Cu-miRNA) together with *miR398*, *miR397*, *miR408*, and *miR857* [37]. The alignment of *miR528* target sites in the three types of target genes revealed seven consensus sites that could be attributable to conserved targeting (Figure 2A and Supplementary Figure S2A). This is further corroborated by the analysis of degradome sequencing data, which verified the associations between *miR52*8 and the three types of conserved targets (Figure 2B–D).

While some target genes of *miR528* are conserved among monocotyledonous species, others exhibit species specificity and functional diversity. To highlight this aspect, we selected four non-conserved yet functionally significant targets for detailed illustration (Figure 2E–H and Supplementary Table S2). For instance, among the predicted target genes of *osa-miR528*, LOC_Os08g42640 encodes a putative C3HC4-type zinc-finger domain-containing protein that regulates flowering time (Figure 2E). Likewise, *Z. mays zma-miR528* was predicted to target GRMZM2G118312, which encodes a putative galactoside 2-alpha-L-fucosyltransferase (Figure 2F); *S. italica sit-miR528* targets a theobromine synthase gene Seita.4G137600 (Figure 2G); *T. aestivum tae-miR528* was predicted to target a PLATZ transcription factor, Traes_6AS_3CC6C63F9, which mediates leaf growth and senescence in plants (Figure 2H). The above examples indicate that *miR528* targets differ among various monocotyledonous plants, yet all these targets are functionally important. The alignment of all the *miR528* target sites led to the identification of one consensus site in *P. virgatum*; two consensus sites in each of *O. sativa*, *Z. mays*, and *T. aestivum*; and three consensus sites in *S. italica* (Figure 2A and Supplementary Figure S2B–F).

## 3. Role of *miR528* in Plant Growth and Development

### 3.1. Flowering Time

At the transcriptional level, *osa-miR528* is highly expressed concomitant with plant development and is light-inducible with a diurnal rhythm. At the post-transcriptional level, the transcript level of mature *osa-miR528* in rice leaves is fine-tuned by the alternative splicing (AS) and alternative polyadenylation (APA) events of primary *osa-miR528* (pri-*osa-miR528*) [14]. Tissue-specific expression analysis, coupled with 5′ RNA ligase-mediated rapid amplification of cDNA ends (RLM-RACE) analysis, showed that *osa-miR528* targets a conserved C3HC4-type zinc-finger transcription factor gene, which is also known as RED AND FARRED INSENSITIVE 2 (*OsRFI2*). Transgenic overexpression of *osa-miR528* was found to attenuate the expression of *OsRFI2* and promote early flowering under long-day conditions [14]. Further, the natural variations in the *osa-MIR528* promoter led to different expression levels of *osa-miR528* in 50 accessions of wild and cultivated rice, which are associated with variations in binding affinities of the SQUAMOSA PROMOTER-BINDING PROTEIN 7 (OsSPL7) transcription factor, which activates *osa-MIR528* expression. Consequently, a conserved photoperiodic flowering pathway in rice mediated by the SPL7–miR528–RFI2 module was established, which likely contributes to the geographic adaption of rice to grow at different latitudes, corresponding to different photoperiod rhythms [14]. *miR528* has also been proposed to regulate flowering time by modulating secondary metabolism. For instance, in Hemerocallis citrina, it targets the key structural gene PGT1-7 and the transcription factor bZIP-1 to control flavonoid modification and accumulation during the bud-to-flower transition, thereby influencing floral organ development and maturation [39].

### 3.2. Plant Architecture

It is known that *miR528* plays a regulatory role in plant development. Transgenic overexpression of pri-*osa-miR528* in creeping bentgrass (*Agrostis stolonifera* L.), which is an important perennial grass species, led to alterations in plant architecture [16]. Transgenic creeping bentgrass exhibited a distinct dwarf phenotype featuring reduced average length of the internodes from each tiller, thicker leaves, and increased number of vascular bundles and tillers, resulting in a denser, uniform, and lodging-resistant plant architecture, which is highly desired in turf grass [16]. Consistent with this, in *Leymus chinensis*, the CRISPR-associated protein 9 (CRISPR/Cas9)-mediated knockout of *miR528* led to a significant increase in tiller number—on average two more tillers than wild-type plants both 1 and 3 months post-transplantation—resulting in enhanced growth rate and biomass accumulation [40]. Further mechanistic insights come from rice, where *miR528* regulates plant architecture by targeting the D3 gene, a key regulator of abscisic acid (ABA) and gibberellin (GA) biosynthesis; its overexpression represses D3 expression, reducing GA and elevating ABA levels, thereby enhancing tillering and reducing plant height by modulating hormonal homeostasis [41].

### 3.3. Seed Development

In comparison to the aforementioned roles in flowering time and plant architecture, there are more studies that demonstrated the functionality of *miR528* in regulating seed development. In maize, the dynamic expression profiling of miRNAs by Solexa deep sequencing in Zhengdan 958, an elite maize hybrid and cultivated widely in China, revealed that the abundance of *zma-miR528a* and *zma-miR528b* decreased linearly in the grain filling stage; and eight putative target genes of *zma-miR528* were identified, which could be grouped by their diverse functionalities, including oxidoreductase activity, signal transduction, development, post-translational regulation, and stress response [17]. Building on its expression dynamics during seed maturation, subsequent studies further demonstrated that *zma-miR528a* is transcriptionally activated during seed germination by high nitrate and auxin levels through TGACG motif-binding factors 1 and 4 (*TGA1/4*) and Auxin Response Element (AuxRE) in its promoter, thereby promoting its accumulation and facilitating seed germination via the regulation of redox homeostasis and hormonal signaling [42]. In the maize inbred line Chang 7–2 (paternal parent of Zhengdan 958), the potential functional role of *zma-miR528* could also be predicted by its high abundance in the latter stage of nutrient storage during kernel development [43]. Meanwhile, in the maize inbred line B73, *zma-miR528* was highly expressed during seed development [44].

In the rice cultivar Nipponbare, high-throughput sequencing data showed that *osa-miR528* was highly expressed in developing rice grains with 7.5-fold greater expression 6–10 days after pollination (DAP) than 1–5 DAP [45]. In the japonica cultivar Zhonghua 11, *osa-miR528* was highly and preferentially expressed in embryos [46]. The putative target genes of *osa-miR528* have been identified to encode copper-binding proteins and L-ascorbate oxidases (AOs), which are involved in diverse developmental and metabolic processes, such as regulating the apoplastic redox state and modulating plant growth and defense responses [46]. Taken together, these results suggest that *miR528* may play a vital role in plant seed development by regulating the expression of a number of target genes.

### 3.4. Embryogenic Development

During somatic embryogenesis (SE), a high level of *miR528* expression is observed in several plant species, such as rice [47] and maize [48,49]. In rice, *osa-miR528* shows high expression in undifferentiated calli and maintains the cells in the meristematic phase by targeting genes coding copper-containing oxidase enzymes, which results in lower production of lignin and a thinner cell wall [47,50]. In maize, *zma-miR528* is required for embryo dedifferentiation during somatic embryogenesis, and it regulates multiple target mRNAs, including basic helix-loop-helix transcription factors, multidrug and toxic compound extrusion/big embryo, SOD, and PLC, by promoting their degradation, translation inhibition, or both [48,49,51,52].

## 4. Role of *miR528* in Plant Stress Responses

### 4.1. Biotic Stress

It has been suggested that *miR528* may play a role in plant antiviral immune responses by targeting immunity-associated genes [53]. Rice plants that were inoculated with rice stripe virus (RSV) showed reductions in the expression of *miR528* and an increase in the accumulation of AGO1 and Argonaute 18 (AGO18) proteins [18]. As a decoy AGO protein incapable of cleaving target RNAs, AGO18 was able to sequester miR528 and prevent it from its incorporation into RNA-induced silencing complex (RISC) with AGO1 [53]. As a result, the reduced RISC efficiency allowed for higher expression of the AO gene that catalyzes ascorbic acid (AsA) oxidation, leading to higher basal ROS accumulation and enhancement in ROS-mediated resistance against RSV infection [18].

In a subsequent study, the same research team reported that the *miR528*-AO antiviral defense pathway was regulated by the transcription factor SQUAMOSA Promoter-Binding Protein-Like 9 (SPL9), which positively regulated *miR528* expression by binding to the GTAC motifs in the *MIR528* promoter and negatively regulated the antiviral defense in rice, whereby the transgenic lines overexpressing SPL9 showed exacerbated symptoms and higher infection rates than the wild-type (WT), whereas the rice spl9-knockout lines showed alleviated RSV infection compared with the WT [54]. Building on these findings, a recently proposed regulatory model further describes a complete copper (Cu)–SPL9–*miR528*–AO–ROS–antiviral pathway in rice, in which virus infection induces copper transporter gene expression and leaf copper accumulation, thereby inhibiting the SPL9-mediated transcriptional activation of *miR528*, leading to the upregulation of its target gene AO, enhanced ROS production, and ultimately increased antiviral defense capacity [55]. It is, therefore, envisaged that the genetic manipulation of *miR528*-mediated gene regulation may impart new and effective tactics to enhance pathogen immunity in crop plants.

Beyond viral defense, *miR528* also contributes to insect resistance in rice. Specifically, during brown planthopper (BPH) infestation, the long non-coding RNA (lncRNA) MSTRG.13957.30 is upregulated and functions as a target mimic, binding *osa-miR528* and preventing it from suppressing the AO gene. This regulatory interaction enhances AO expression and activates ascorbic acid-related defense pathways, thereby contributing to BPH resistance [56].

Furthermore, *miR528* has been shown to function in cross-kingdom immune regulation. In maize, *zma-miR528b-5p* directly targets the fungal virulence gene FvTTP in Fusarium verticillioides, reducing fumonisin B production and promoting salicylic acid (SA) accumulation and pathogenesis-related protein 1 (PR1) expression, ultimately reinforcing SA-mediated immune responses in the host plant [57].

### 4.2. Salt Stress

The role of *miR528* in response to salt stress was investigated in creeping bentgrass where *miR528* was significantly induced by salt stress [16], which was corroborated by similar observations in maize [58]. Upon salt treatment, the overexpression of rice *osa-miR528* in creeping bentgrass directly repressed the expression of its target genes AAO and copper-ion-binding protein 1 (CBP1), which mediated oxidation homeostasis and alleviated cellular damage by salt stress. It was postulated that *miR528* may also positively regulate the genes involved in some other signaling pathways, such as a high-affinity K transporter (HAK5) and a salt stress-induced transcription factor (NAC60), induce the activity of antioxidant enzymes such as catalase (CAT) to maintain ROS homeostasis, and interact with other stress-related *miRNAs* to form a regulatory network to coordinately integrate multiple stress regulators in response to salt stress [16]. Consistently with these findings, in rice, the salt-induced upregulation of *miR528* was shown to suppress the expression of its target gene AO, thereby increasing the accumulation of AsA and ABA, which subsequently promoted proline synthesis, enhanced osmotic adjustment and ROS scavenging, and ultimately improved salt tolerance [59].

### 4.3. Temperature Stress

Temperature stress is one of the major environmental factors that affects not only plant growth but also fruit postharvest shelf life and quality. Using deep sequencing and bioinformatic and molecular analyses, in banana, the expression of *mac-miR528* was severely suppressed by low temperature but significantly induced by heat stress [19]. The opposite effect was observed in switchgrass, where *miR528* was significantly attenuated by heat stress [60]. Also, in banana, degradome sequencing analysis revealed that miR528 acts in concert with its corresponding target gene (PPO), which plays an imperative role in regulating plant response to temperature stress [19]. Further supporting these findings, exogenous melatonin treatment upregulates *miR528* expression in banana, which represses its target gene *MaPPOs*, thereby reducing PPO activity, enhancing peroxidase (POD) and CAT activities, maintaining ROS homeostasis, and ultimately improving cold tolerance and delaying peel browning [61]. In rice, the co-overexpression of *osa-miR528*, *osa-miR397*, and *osa-miR408* synergistically enhances cold tolerance by reducing leaf damage, ion leakage, and malondialdehyde (MDA) accumulation under low-temperature stress [62]. Consistently, in the cold-tolerant japonica rice variety JL (Jilin Sunset), *osa-miR528* was significantly downregulated under cold stress, leading to the de-repression of its target gene Os03g0152000, enhanced antioxidant capacity, and improved cold tolerance [63]. *MiR528* plays an important regulatory role in plant responses to low-temperature stress by modulating redox balance and maintaining cellular homeostasis. However, its specific regulatory mechanisms remain unclear and warrant further investigation.

### 4.4. Water Stress

It has been recognized that *miR528* could mediate post-transcriptional regulation in response to water stress, which is a major constraint for crop yield improvements. In wild emmer wheat (*Triticum turgidum* ssp. *dicoccoides*), the ancestor of domesticated durum wheat (*T. turgidum* ssp. *durum*), the expression of *miR528* was significantly downregulated in leaf and root tissues by drought stress [20]. Likewise, in sugarcane (*Saccharum* spp.), *ssp-miR528* was downregulated in both sugarcane cultivars RB867515 (high tolerance to drought) and RB855536 (low tolerance to drought) on the fourth day of drought stress treatment, which is suggestive of its role in reducing growth in response to drought stress [64]. When the seedlings of three maize inbred lines (Hz32, B73, and Mo17) with different sensitivities to waterlogging were assayed under controlled experimental conditions, it was found that *miR528* modulates root development as a key regulator in the post-transcriptional regulatory system in response to short-term waterlogging stress [65]. Further supporting its functional versatility, the overexpression of *osa-miR528-3p* in rice enhances drought tolerance by increasing indole-3-acetic acid (IAA) levels and reducing ROS accumulation, thereby promoting root and leaf elongation, as well as biomass accumulation [66]. Furthermore, in *Agropyron mongolicum*, *miR528* is downregulated under drought stress, leading to the upregulation of its target gene HOX24, a transcription factor involved in ABA signaling and water deficit response, thereby suggesting a regulatory role of miR528 in drought tolerance via modulation of HOX24-mediated pathways [67]. In rice, *osa-miR528* displays differential expression under drought stress in the jointing stage across varieties, with notably altered levels in the drought-sensitive cultivars IR64 and Kitaake, indicating its potential role as a key regulatory node in mediating reproductive-stage drought responses [68]. In line with these findings, in the drought-tolerant rice variety Azucena, the drought-induced downregulation of *miR528* enhances drought tolerance by increasing the activities of antioxidant enzymes such as SOD and peroxidase (POX), reducing ROS accumulation and improving root tip cell viability and elongation capacity [69]. Collectively, these findings indicate that *miR528* regulates both hormonal signaling and antioxidant defense pathways, thereby acting as a central hub in plant drought stress responses.

### 4.5. Arsenic (As) Stress

Arsenic (As), which is ubiquitous and abundant in the earth’s crust, is a nonessential metalloid and very toxic to plants. Arsenite [As (III)] and arsenate [As (V)] are the predominant inorganic species of As in soil and are readily interconvertible depending upon soil changing redox potential and pH [70]. Rice is highly efficient in As uptake, particularly in As-contaminated soils, relative to other cereals, such as barley and wheat [71]. In rice, the analysis of miRNA expression pattern under As (III) stress at the genome-wide level by using a high-throughput sequencing strategy and stem-loop real-time quantitative PCR revealed that *osa-miR528* was strongly upregulated by As (III) in the roots of Minghui 86, a rice cultivar that is sensitive to As (III) stress [72]. It is hypothesized that in the proposed network of arsenite-responsive miRNAs, the upregulation of *miR528* in the roots of rice seedlings in response to As stress downregulate the copper-ion-binding protein (CBP) and IAA-alanine resistance protein 1 (IAR1) genes, to save copper for the most essential functions and keep most IAA in conjugated forms, respectively, thereby protecting rice seedlings from As (III) damage [72].

The functional role of *osa-miR528* in As (III) tolerance was directly demonstrated by overexpressing *osa-miR528* (*Ubi::MIR528*) in a japonica rice Nipponbare cultivar, which is more tolerant to As(III) than Minghui 86 [21]. Compared with WT plants, *Ubi::MIR528* plants presented highly upregulated expression of *osa-miR528* in both the roots and leaves and showed As (III) sensitivity under stress conditions, which was likely due to the strong alteration in antioxidant enzyme activity and amino acid profiles and the impairment of the As (III) uptake, translocation, and tolerance systems of rice [21]. It is of interest to note that *osa-miR528* exhibited opposite expression patterns in response to As (III) and As (V) exposure; specifically, *osa-miR528* was upregulated upon As (III) stress but downregulated upon As (V) stress [73].

### 4.6. Heavy-Metal Stress

In addition to As stress, heavy metals represent a class of looming soil contaminants and environmental pollutants, and aluminum (Al) and cadmium (Cd), in particular, are extremely toxic to plants [74,75]. There is a growing body of evidence that *miR528* plays a crucial regulatory role in the processes of heavy-metal stress response and/or tolerance. In rice, *osa-miR528*, which putatively targets an F-box/LRR repeat gene, MORE AXILLARY GROWTH 2 (*MAX2*), was strongly downregulated in rice seedling roots under Al stress [76]. Microarray-based analysis showed that upon Cd exposure, *osa-miR528* was upregulated in rice seedling roots, and metal-responsive element (MRE) was identified in the promoter region of *osa-MIR528*, which lends further credence to the potential involvement of *osa-miR528* in regulating Cd stress tolerance and highlights *miR528* as an area of interest for further investigation into heavy-metal tolerance in plants [77]. Beyond transcriptional changes, further studies have demonstrated that *miR528* modulates ROS homeostasis, which is critical to mitigating heavy-metal-induced oxidative stress. Specifically, under Cd stress, rice *miR528* represses the expression of its target gene unknown copper-binding-like protein 23 (*UCL23*), a small copper protein involved in ROS accumulation, thereby reducing oxidative stress; meanwhile, *miR528* is negatively regulated by the transcription factor WRKY transcription factor 51 (*WRKY51*), forming a *WRKY51–miR528–UCL23* regulatory module that coordinates the rice response to Cd toxicity [78]. Similarly, Under chromium stress, the downregulation of *zma-miR528* alleviates its repression on the SOD gene, thereby enhancing ROS scavenging capacity, mitigating oxidative damage, and improving root vitality and chromium stress tolerance, highlighting *miR528* as a pivotal regulator in the antioxidant defense mechanism [79]. Furthermore, *miR528* may mediate spatially distinct growth responses under heavy-metal stress. In maize, in response to Al stress, the accumulation of *zma-miR528* was induced in primary roots but reduced in seminal roots, suggesting a role of *zma-miR528* in mediating the differential growth responses to Al stress [23].

### 4.7. Nitrogen Homeostasis

There is growing recognition that *miR528* plays an important role in the plant response to deficiencies in plant nutrients, such as nitrogen (N) and zinc (Zn), by modulating nutrient uptake, phloem-mediated long-distance transport, and nutrient homeostasis. It has been suggested that *miR528* may act as a systemic signal in coordinating plants’ responsive physiological activities to alleviate nutrient deficiency stresses or toxicities [24]. In *creeping bentgrass*, *miR528* was significantly suppressed under N deprivation, and overexpressing rice *pri-miR528* in *creeping bentgrass* plants resulted in a significant enhancement in tolerance to N deficiency stress, which was associated with increases in biomass, total N accumulation and chlorophyll synthesis, reduction in AAO activity, and increases in NITRITE REDUCTASE (NiR) activity [16]. On the other hand, applying excessive quantities of chemical N fertilizer, coupled with a decrease in N use efficiency, resulted in both looming environmental problems and yield losses due to lodging under N-luxury conditions [80]. In maize, *zma-miR528* was found to affect lignin content and composition and ameliorate lodging resistance in maize seedlings under N-luxury conditions by negatively regulating the expression of the ZmLACCASE3 (*ZmLAC3*) and ZmLACCASE5 (*ZmLAC5*) genes [81]. Building on this, recent studies have further revealed that under varying nitrogen conditions, *zma-miR528* modulates Casparian strip formation by targeting the lignin-associated gene *ZmLAC3*, thereby influencing root apoplastic barrier development and ion homeostasis, and uncovering a novel regulatory link between nitrogen signaling and nutrient transport in maize [82].

## 5. Conclusions and Perspectives

Overall, as a monocot-specific miRNA in plants, *miR528* has drawn considerable research interest in recent years because of its involvement in plant growth and development and in response to numerous biotic and abiotic stress conditions. There is growing recognition that *miR528* acts in concert with many other miRNAs that have been found to be co-responsive to certain stress factors or targeting the same genes. As comprehensively summarized in Table 2, *miR528* regulates diverse agronomic traits across major monocot crops, highlighting its potential as a key target for crop improvement strategies. However, the formation and evolution of the potentially monocot-specific regulatory networks in which *miR528* participates are intriguing but remain elusive and deserve further investigation. A comprehensive understanding on the *miR528*-mediated gene regulatory networks in plant growth, development, and stress response cascades will facilitate the development of new strategies aimed at improving crop resilience and production.

## Figures and Tables

**Table 1:** The summary of *MIR528* from 17 monocot plant species.

Locus ID	Organism	Genome Location ^a^	Overlapping Gene ^b^
*aof-MIR528*	*Asparagus officinalis* L.	NC_033797.1:10579184~10579302,+	×
*bdi-MIR528*	*Brachypodium distachyon* (L.) P.Beauv.	Bd1:73295140~73295266,−	BRADI_1g76465v3
*mac-MIR528*	Musa acuminata Colla	Chr8:10341398~10341507,−	GSMUA_Achr8G13592_001
*ogl-MIR528*	Oryza glaberrima Steud.	GL455988.1:1549995~1550120,+	ORGLA03G0021200
*oni-MIR528*	Oryza nivara S.D.Sharma and Shastry	Chr3:1341691~1341816,+	ONIVA03G01960
*oru-MIR528*	Oryza rufipogon Griff.	HG417167.1:1412714~1412839,+	ORUFI03G02090
*osa-MIR528*	*Oryza sativa* L.	Chr3:1667310~1667435,+	LOC_Os03g03724
*pha-MIR528*	Panicum hallii Vasey	Chr09:69425755~69425882,−	×
*pvi-MIR528a*	*Panicum virgatum* L.	Chr01N:69192314~69192445,+	Pavir.Aa02527.1
*pvi-MIR528b*	*Panicum virgatum* L.	Chr09K:87418444~87418571,−	×
*pvi-MIR528c*	*Panicum virgatum* L.	Chr09N:120070864~120070991,−	×
*pap-MIR528*	Phalaenopsis aphrodite Rchb.f.	NEWO01000071.1:538724~538838,+	×
*pda-MIR528*	*Phoenix dactylifera* L.	NW_008247750.1:18899~19050,−	×
*scu-MIR528*	*Saccharum cultivar*	JXQF01030556.1:160~293,+	×
*sit-MIR528*	*Setaria italica* (L.) P. Beauvois	Scaffold_9:57157041~57157170,−	SETIT_040610mg
*sbi-MIR528*	*Sorghum bicolor* (L.) Moench	Chr1:79165414~79165537,−	sbi-MIR528
*spo-MIR528*	*Spirodela polyrhiza* (L.) Schleid.	Pseudo17:196564~196705,−	×
*tae-MIR528a*	*Triticum aestivum* L.	Chr4B:162371969~162372094,+	×
*tae-MIR528b*	*Triticum aestivum* L.	Chr4D:32161888~32162013,+	×
*tae-MIR528c*	*Triticum aestivum* L.	Chr5A:210090100~210090222,+	×
*zma-MIR528a*	*Zea mays* L.	Chr1:6409229~6409390,+	zma-MIR528a
*zma-MIR528b*	*Zea mays* L.	Chr9:153752320~153752436,−	zma-MIR528b

^a^ ‘+’ and ‘−’ indicate the forward and reverse strands, respectively. ^b^ ‘×’ indicates nonexistence.

**Table 2:** *MiR528*-regulated agronomic traits and potential applications in monocot crops.

Regulated Trait	Crop Species	Experimental Approach	Regulatory Mechanism and Trait Outcomes	Potential Application	Reference
Flowering time	Rice	Overexpression	Represses *OsRFI*2 via *OsSPL7* activation, promoting early flowering under long-day conditions	Photoperiod adaptation and regional cultivation optimization	[14]
Plant architecture	*Creeping bentgrass*	Transgenic overexpression of pri*-osa-miR528*	Reduces internode length, increases tillering and vascular bundles, and enhances lodging resistance	Breeding for lodging-resistant cultivars	[16]
Seed development	Rice	Expression profiling	Targets copper-binding proteins and ascorbate oxidases during grain filling	Enhancing grain development and seed vigor	[45,46]
Embryogenic development	Maize	Functional validation	Regulates SOD, PLC, and transcription factors to promote somatic embryogenesis	Optimization of somatic embryogenesis and plant regeneration protocols	[48,49]
Biotic stress (viral)	Rice	Mutant analysis	AGO18 sequestration de-represses AO, increasing ROS-mediated antiviral defense	Breeding virus-resistant varieties	[18,54]
Biotic stress (insect)	Rice	Expression analysis	lncRNA-mediated AO de-repression activates ascorbate defense against brown planthopper	Development of brown planthopper-resistant cultivars	[56]
Biotic stress (fungal)	Maize	Cross-kingdom study	Targets fungal FvTTP to reduce mycotoxins and enhance SA signaling	Breeding Fusarium-resistant maize with reduced mycotoxin accumulation	[57]
Salt stress	*Creeping bentgrass*	Transgenic overexpression of pri*-osa-miR528*	Suppresses AAO and CBP1, improving ion homeostasis	Breeding salt-tolerant cultivars	[16]
Salt stress	Rice	Overexpression	AO suppression elevates AsA/ABA, enhancing osmotic adjustment and ROS scavenging	Improving salt tolerance via ABA–AsA–ROS modulation	[59]
Temperature stress (cold)	Banana	Melatonin treatment	*MaPPOs* repression reduces enzymatic browning, enhances antioxidant activity	Postharvest preservation via PPO suppression	[61]
Temperature stress (cold)	Rice	Co-overexpression	Synergistic action with *miR397/miR408* reduces oxidative damage	Developing cold-tolerant rice through miRNA synergy	[62]
Water stress (drought)	Wheat	Expression profiling	Downregulation coordinates drought-responsive gene networks	Breeding drought-resilient wheat cultivars	[20]
Water stress (drought)	Rice	Overexpression	Increases IAA accumulation and reduces ROS, promoting root elongation	Enhancing drought tolerance via IAA and ROS regulation	[66]
Arsenic (As III) stress	Rice	Overexpression	Suppresses CBP and IAR1, disrupting As uptake and antioxidant defense	Enhancing arsenic tolerance and reducing arsenic accumulation	[21]
Heavy-metal stress (Al)	Maize	Tissue-specific profiling	Mediates root-specific responses to aluminum toxicity	Enhancing aluminum tolerance in maize via root-specific regulation	[23]
Heavy-metal stress (Cd)	Rice	Regulatory module analysis	*WRKY51-miR528-UCL23* axis coordinates ROS homeostasis under cadmium stress	Mitigating cadmium toxicity by modulating ROS homeostasis	[78]
Nitrogen homeostasis	Maize	Functional characterization	Targets *ZmLAC3* to modify lignin biosynthesis and Casparian strip formation	Improving nitrogen use efficiency	[81,82]
Nitrogen homeostasis	*Creeping bentgrass*	Transgenic overexpression of pri*-osa-miR528*	Enhances N assimilation via increased NiR activity and chlorophyll synthesis	Enhancing nitrogen assimilation and growth under deficiency	[16]

## Data Availability

No new data were created in this study. Data sharing is not applicable to this article.

## Supplementary Materials

The following supporting information can be downloaded at: https://www.mdpi.com/article/10.3390/ijms26157334/s1.
