# Supplementary material for: MicroRNA528 and Its Regulatory Roles in Monocotyledonous Plants
Source: Int J Mol Sci. 2025 Jul 29;26(15):7334. doi: 10.3390/ijms26157334 (PMC12347108; doi:10.3390/ijms26157334)
Supplement: Supplementary file 1 [file ijms-26-07334-s001.zip › Supplementary Materials.pdf]

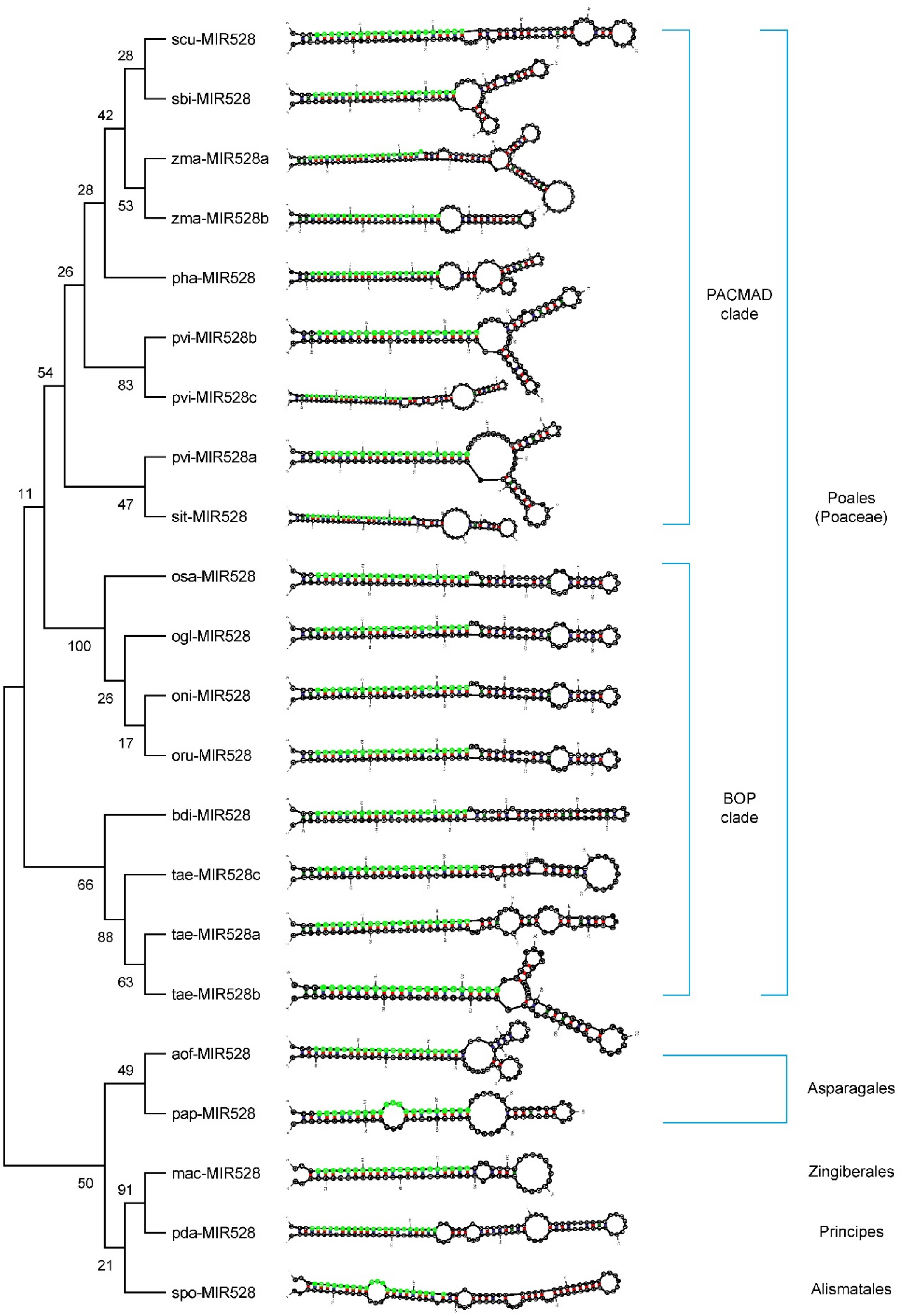

Figure S1. The maximum parsimony (MP) tree reconstructed with precursors of miR528 using MEGA, with 100 bootstrap replicates; bootstrap support values are shown at the nodes. The stem-loop structure of MIR528 was predicted using the Mfold web server (http://unafold.rna.albany.edu/?q=mfold). The mature sequence was highlighted by green.

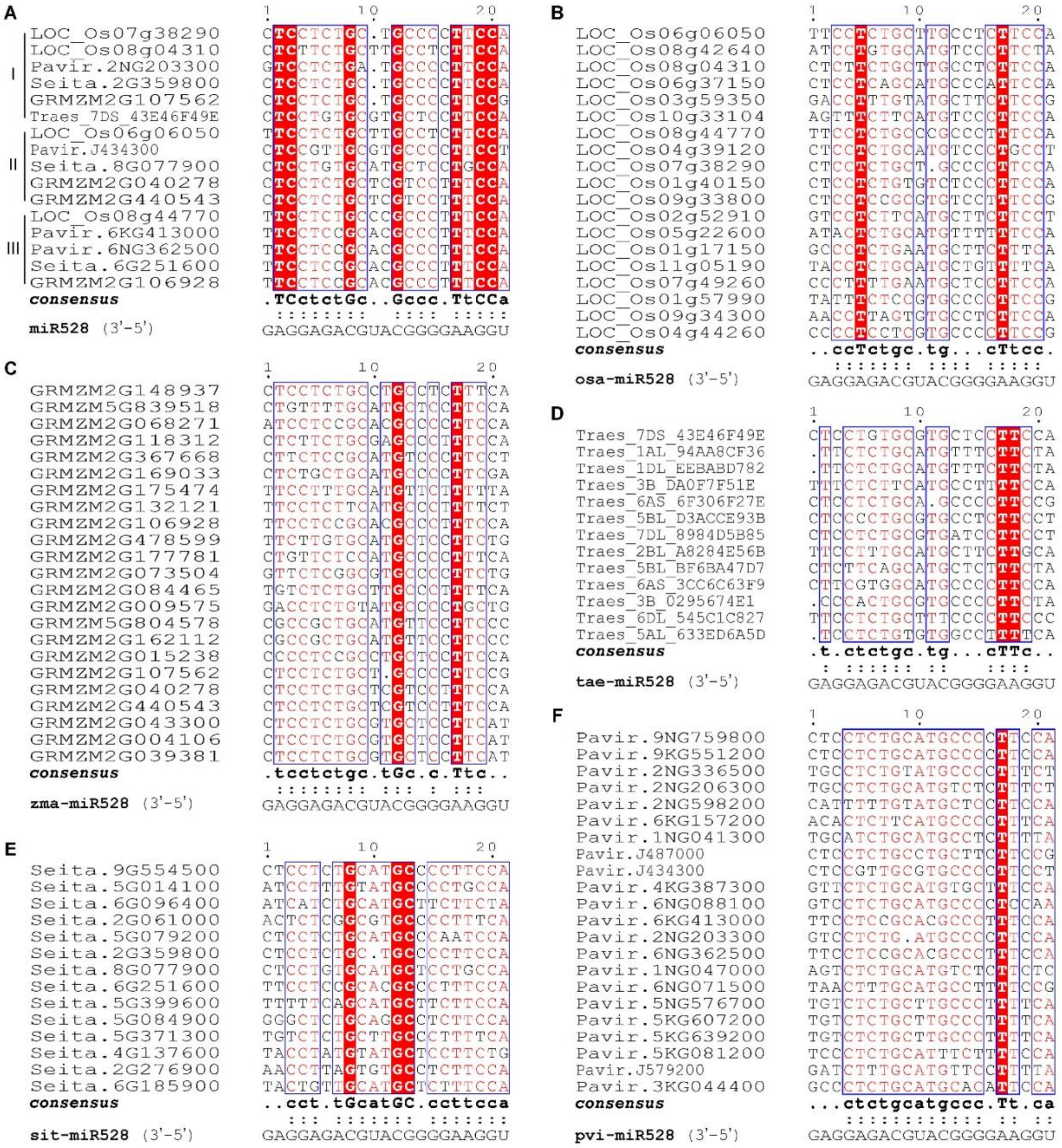

Figure S2. (A) Alignment of miR528 target sites of conserved target genes from five different plants. The five plants were as follows: Oryza sativa L. (osa), Panicum virgatum L. (pvi), Setaria italica (L.) P. Beauvois (sit), Triticum aestivum L. (tae) and Zea mays L. (zma). The target genes of miR528 were predicted by searching the transcripts for complementary sequences using the psRNATarget server with a more stringent cut-off threshold: maximum expectation (E) = 3.0 (http://plantgrn.noble.org/psRNATarget/home). The target site sequences of miR528 were aligned using the ClustalX program, and presented using the ESPript 3.0 with default sequence similarities depiction parameters (http://espript.ibcp.fr/ESPript/ESPript/index.php). Group I to III are the target genes encoding the plastocyanin-like domain containing proteins, the F-box domain and LRR containing proteins, and the copper/zinc superoxide dismutases, respectively. (B~F) Sequence alignments of miR528 target sites of diverse target genes from O. sativa (B), Z. mays (C),T. aestivum (D), S. italica (E) and P. virgatum (F).
